# Determinants of compliance to the facemask directive in Greece: A population study

**DOI:** 10.1371/journal.pone.0248929

**Published:** 2021-03-19

**Authors:** Georgios Labiris, Eirini-Kanella Panagiotopoulou, Asli Perente, Eleftherios Chatzimichael, Ioannis Fotiadis, Sergios Taliantzis, Aristeidis Konstantinidis, Doukas Dardabounis

**Affiliations:** Department of Ophthalmology, University Hospital of Alexandroupolis, Dragana, Alexandroupolis, Greece; Middlesex University, UNITED KINGDOM

## Abstract

**Purpose:**

Primary objective of this study was to identify potential difficulties and/or discomfort when using a facemask. Moreover, to explore the impact of spectacles, contact lenses and visual acuity on the compliance to the facemask directive.

**Methods:**

This is a prospective study that was conducted at the Department of Ophthalmology, University Hospital of Alexandroupolis, Greece between June 2020 and August 2020. Greek speaking citizens with permanent residency in Greece above 18 years old were included. A custom questionnaire (DeMask-20) was constructed and validated, which pertained to the perceived difficulty and discomfort when using a facemask. It contained 20 items grouped in 8 subscales (driving, near vision, distance vision, ocular discomfort, role limitation, collaboration, dependency on others, emotional stress). Perceived difficulty and discomfort when using a facemask, compliance and correlations of compliance with DeMask-20 scores, demographics, spectacle and/or contact lens use, and visual acuity were evaluated.

**Results:**

The number of factors was determined through factor analysis. Cronbach’s alpha ranged from 0.716 for the “Role limitation” subscale to 0.938 for “Ocular discomfort” subscale. 1,214 participants (402 men, 812 women, mean age 36.79±12.50 years) completed the DeMask-20 instrument. Mean DeMask-20 score of all study participants was 3.79±0.71. Significant differences in DeMask-20 score were detected in gender (p = 0.009), spectacle use (p = 0.034), contact lens use (p = 0.049), and binocular distance visual acuity (bDVA) (p = 0.001). Mean compliance of all participants was 4.05±0.96. Men, people <50 years and spectacle wearers showed significantly worse compliance (p<0.05). Moreover, professional workers and professional drivers demonstrated significantly better compliance (p = 0.008 and p = 0.047). Significant correlation was detected between compliance and DeMask-20 score (p<0.001, R^2^ = 0.471). Significant correlations were detected with driving, near vision, distance vision, collaboration, role limitation, emotional stress (p<0.05, R^2^: 0.386–0.493).

**Conclusions:**

Factor analysis suggested that the DeMask-20 instrument demonstrates adequate validity, while Cronbach’s alpha indicated sufficient internal consistency of all subscales. This study provided the necessary methods that could evaluate compliance trends and the efficacy of healthcare interventions against COVID-19. Our outcomes suggest that young males who use spectacles should be targeted by Greek Healthcare authorities in order to improve compliance rates.

## Introduction

Coronavirus disease 2019 (COVID-19) caused by severe acute respiratory syndrome coronavirus 2 (SARS-CoV-2), was first described in December 2019 after the diagnosis of several cases with community-acquired pneumonia of unknown etiology in Wuhan city of China [1, 2]. Since then, COVID-19 was rapidly spread all around the world leading to 41.3 million infections and more than 1,133,000 deaths until 21th of October 2020 [3]. On 11th of March 2020, the World Health Organization (WHO) declared COVID-19 as the second pandemic of 21^st^ century. To restrict the transmission of this highly contagious disease, governments have implemented drastic measures including border and school closure, quarantine and limitation of all socioeconomic activities to only essentials [1]. Behavioral and social sciences can also support the negative effects of this pandemic [4]. Hand hygiene, facemask use and social distancing of at least 2 meters are among the most important personal protective measures [1, 5–8]. It is worth mentioning that until recently there was controversy regarding the beneficial impact of facemask use by the general public [1, 6, 7, 9–11]. Although the use of facemasks from healthcare workers is generally accepted, there is a great debate regarding the necessity of facemask usage from general public [1, 6, 7]. Among the supporters of this aspect, the prevailing theory is that facemasks increase the persons’ sense of security reducing thus their compliance to other protective measures [7, 9, 12]. However, according to the latest directive of the WHO [9], facemask use is strongly advised for the general public even for outdoor activities.

Several studies dealt with the protective effect of facemask use during this pandemic. Eikenberry et al. developed a mathematical model using the data from New York and Washington regarding the transmission of COVID-19. They concluded that the wide use of facemasks from general public decreases the transmission rate, especially if it is accompanied with other recommended hygiene measures [6]. Javid and his colleagues reported that 40–80% of SARS-CoV-2 transmissions occur from healthy asymptomatic individuals; therefore, they supported that wide use of facemasks may be of paramount importance [7].

Despite the aforementioned studies, there have also been reported different facemask-related problems including facemask-induced dermatitis, retroauricular dermatitis due to ear loop facemasks and headaches [13, 14]. Morishima et al. evaluated the awareness of problems while wearing facemask in 2009, 2012, and 2015 using a questionnaire. The most frequent problems for men were humidity in the facemask, blurring of glasses and breathing difficulty. Women reported exactly the same problems with the makeup removing to be an additional complaint [15]. It is worth mentioning that, during the study period (June—August 2020), the incidence of COVID-19 in Greece was one of the lowest among other countries of the European Union and the European Economic Area [16]. More specifically, in June 2020 there were 500 new cases with the number of deaths during this month to be 9, while, in August, these numbers increased significantly leading to 5088 new cases and 61 new deaths from COVID-19 until the end of this month [17]. Regarding facemask use directives during the period of study, it was mandatory for healthcare providers, professional drivers and passengers, for workers and general public at airports and the shop staff. As the number of cases was increasing, the authorities widened these measures, therefore from 10th August 2020 facemask usage was obligatory in places of worship, supermarket and shops for both customers and staff [18–20].

Within this context, the primary objective of this study was to identify potential difficulties and/or discomfort when using a facemask, evaluate the impact of spectacles, contact lenses and visual acuity, and explore their impact on compliance to the directive on facemask use.

## Materials and methods

### Setting

This is a prospective study. Study protocol adhered to the tenets of the Declaration of Helsinki. The institutional review board of Democritus University of Thrace approved the study protocol. The study was conducted at the Department of Ophthalmology in the University Hospital of Alexandroupolis, Greece, between June 2020 and August 2020. Official registration number of the study is NCT04501172 (https://clinicaltrials.gov/ct2/show/NCT04501172)

### Participants

Participants were contacted through social networks (Facebook) with a link to an online questionnaire. A cover letter describing the scope and eligibility criteria of the questionnaire accompanied the link. The online questionnaire was open for one month (June 2020 to July 2020). Eligibility criteria, which were described in detail in the cover letter, included age above 18 years old, adequate literacy of Greek language and permanent residence in Greece. Questionnaires completed by people younger than 18 years were excluded from data analysis. All other questionnaires were considered to meet the inclusion criteria and continued for further analysis.

### The DeMask-20 instrument

Literature review on a validated instrument regarding facemask-wearing trends for Greek speaking populations returned no results. Thus, an exploratory interview study was designed to create the baseline for a questionnaire development. A panel consisting of 2 ophthalmologists, 2 nurses with experience in ophthalmology outpatient care, and a psychologist were recruited for the exploratory study. A number of items covering attitudes on facemask wearing were summarized and written as interview questions. Individual interviews with 10 participants who had no previous contact with any of the members of the panel took place. The interviews were analyzed and the findings served as the basis for identifying the variables of interest that would be operationalized in specific items (questions) to be used in our instrument.

The final version of the questionnaire consisted of 2 parts. The first part pertained to the participant’s demographic characteristics, with items regarding age, reported binocular distance visual acuity (bDVA), spectacle and contact lens use, potential health vulnerability [21], and compliance to the facemask wearing directive. For the enrolment in the health vulnerable group, study participant had to meet one of the following criteria: age of 65 years or older, severe heart or respiratory disease, resistant hypertension, uncontrolled diabetes mellitus, severe neurological or neuromuscular disease, kidney or liver failure, high body mass index (BMI), cancer, immunodeficiency or pregnancy [21].

The second part of the instrument consisted of twenty items that constructed 8 subscales and pertained to the potential difficulty and/or discomfort when using a facemask: DeMask-20 subscales were: a) driving (2 items), b) near vision (5 items), c) distance vision (3 items), d) ocular discomfort (3 items), e) role limitation (3 items), f) collaboration (1 item), g) dependency on others (2 items), and h) emotional stress (1 item). The 6-category ordinal polytomous items I1 to I11 were transformed to 5-category Likert-scale items for easier data interpretation and data analysis. Specifically, the categories “a) I need to remove my facemask” and “b) I have almost stopped this activity because of my vision and the use of a facemask” of the original item-version were merged into the category “1 = significant difficulty/discomfort”. The numbering of the rest categories was converted accordingly (c → 2 = great difficulty, d → 3 = some difficulty, e → 4 = little difficulty, f → 5 = no difficulty). On the other hand, the original I12 to I20 were 5-category Likert scale items (1 = absolutely agree, 5 = absolutely disagree) and no conversion was necessary. A total DeMask-20 score for each participant was obtained from the average of all subscales. Items I1–I5 were optional (I1, I2 were addressed to professional drivers, and I3–I5 to professional workers), while items about demographic characteristics and I6–I20 were mandatory and had to be answered to allow the online questionnaire to be submitted.

### Statistical analysis

Construct validity of the questionnaire was evaluated by exploratory factor analysis (EFA). As extraction method, Principal Component Analysis (PCA) was applied because it is one of the most simplified and commonly used methods of EFA. Initially, we used an eigenvalue (EV) > 1 (Kaiser’s criterion) to determine the number of factors, in combination with a scree plot. To determine whether the data were adequate for factor analysis (FA), the Kaiser-Meyer-Olkin (KMO) measure was calculated. KMO scores between 0.8 and 1 indicate the appropriateness of the sample for FA. In addition, the Bartlett’s test of sphericity was calculated. If the test was significant (p < 0.001), the data were suitable for FA [22]. Finally, since we expected that underlying factors may be related, we used oblique rotation (direct oblimin) to optimise configuration on factors (Delta = 0) [23, 24]. Items were considered loaded onto a factor if values exceeded 0.40 and were considered uniquely loaded if cross-loadings on other factors were less than 0.40 [23, 24]. After the number of factors had been determined, the internal consistency of DeMask-20 subscales was evaluated by Cronbach’s alpha (a) estimation.

Data distribution of the questionnaire items was tested with Shapiro-Wilk test. Between-group comparisons of data for which the hypothesis of normality is satisfied were made using independent samples Student’s t-test or one-way ANOVA. Data for which the hypothesis of normality is not satisfied were assessed with Mann–Whitney U test or Kruskal–Wallis H test. P-values lower than 0.05 were considered statistically significant. All statistical analyses were performed with SPSS Statistics for Windows software (version 20.0, IBM Corp.)

## Results

### Construct validity and reliability

**Fig 1:**
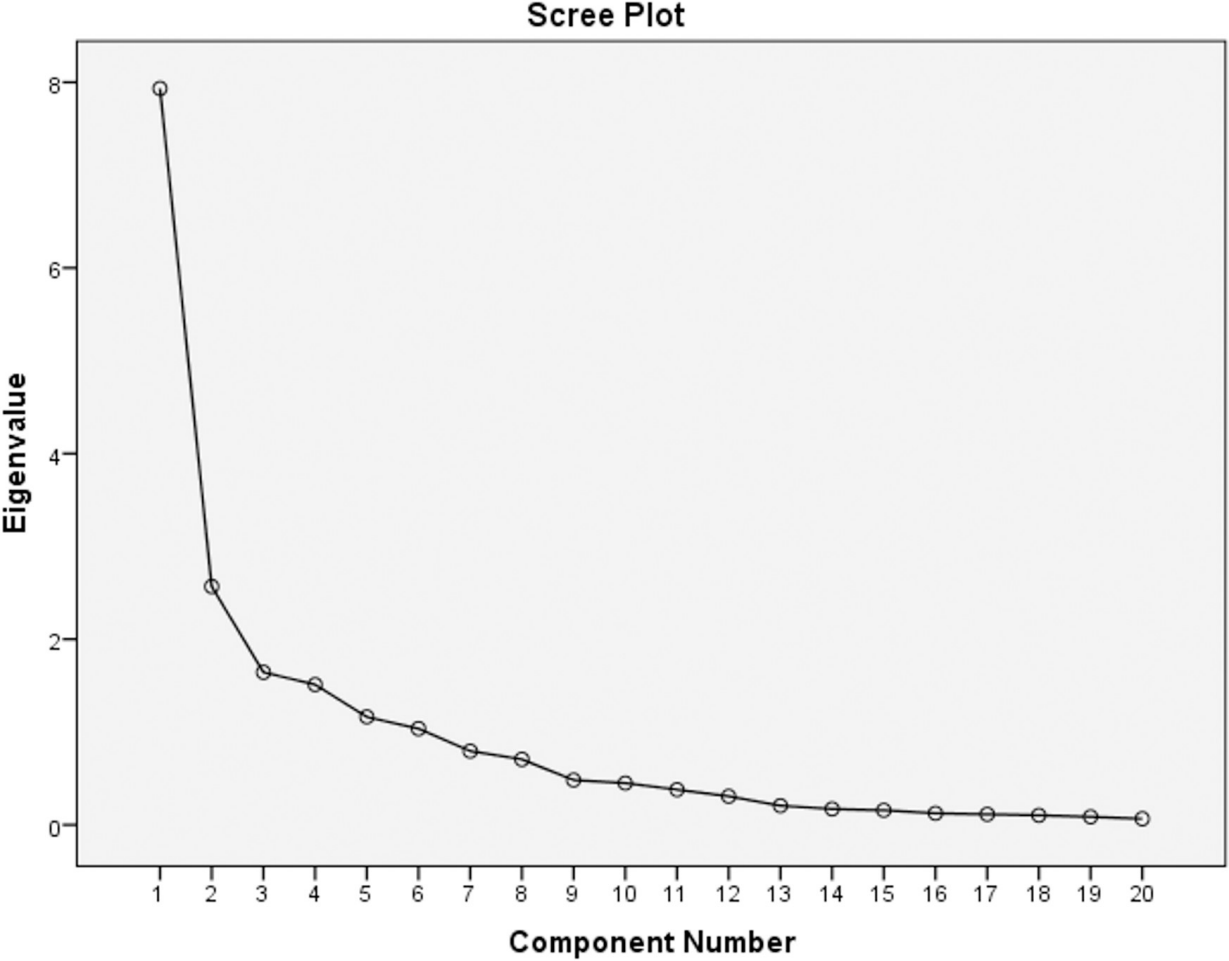
Scree plot of the eigenvalues of the factors after factor analysis. Six factors with eigenvalue > 1.0 were found.

Factor analysis revealed six factors with EV > 1: EV_factor 1_ = 7.933, EV_factor 2_ = 2.568, EV_factor 3_ = 1.644, EV_factor 4_ = 1.511, EV_factor 5_ = 1.163, and EV_factor 6_ = 1.036, which explained 79.27% of the variance of the items (Fig 1). The Kaiser-Meyer-Olkin (KMO) measure, representing the sampling adequacy for the analysis, was 0.835. The Bartlett’s test of sphericity was significant (p < 0.0001), rejecting the null hypothesis that our items are uncorrelated, and indicating that FA would be useful as a data reduction technique. The pattern matrix (Table 1) demonstrates the items that are loaded to each factor after rotation. All loading values of the items were above 0.7. Item I17 was not included in the subscale “Ocular discomfort”, which included items I18, I19, I20, due to its low loading value (0.496) and was evaluated as a distinct subscale which contained a single item. The same was applied for item I5 that demonstrated low loading values to several factors.

**Table 1:** Factor loadings of the DeMask-20 instrument pattern matrix after rotation.

Rotated Component Matrix^a^							
Subscales	Items	Component					
		1	2	3	4	5	6
Near vision	I9	0.904					
	I3	0.863					
	11	0.848					
	I4	0.809					
	I10	0.759					
Ocular discomfort	I20		0.925				
	I18		0.911				
	I19		0.884				
Emotional stress	I17		0.496				
Distance vision	I7			0.831			
	I8			0.830			
	I6			0.759			
Collaboration	I5			0.410	0.311	0.360	
Dependency	I16				0.932		
	I15				0.896		
Driving	I2					0.868	
	I1					0.846	
Role limitation	I12						0.765
	I13						0.761
	I14						0.716

Extraction Method: Principal Component Analysis.

Rotation Method: Oblimin with Kaiser Normalization.

a. Rotation converged in 8 iterations.

Reliability analysis was done by Cronbach’s alpha estimation as an index of internal consistency for each subscale (Table 2) [25]. Cronbach’s alpha ranged from 0.716 for the “Role limitation” subscale to 0.938 for “Ocular discomfort” subscale. Thus, it becomes obvious that the majority of the subscales presented high internal consistency.

**Table 2:** Reliability analysis of DeMask-20 instrument.

Subscales	Number of items	Items	Cronbach’s alpha	95% lower confidence limit
Driving	2	I1, I2	0.9349	0.9144
Near vision	5	I3, I4, I9, I10, I11	0.937	0.9276
Collaboration	1	I5	NA	NA
Distance vision	3	I6, I7, I8	0.8328	0.8118
Role limitation	3	I12, I13, I14	0.7162	0.6814
Dependency	2	I15, I16	0.9359	0.9266
Emotional stress	1	I17	NA	NA
Ocular discomfort	3	I18, I19, I20	0.9381	0.9305

NA: Not applicable (needs two or more items); I: Item.

### Study outcomes

1,214 participants [402 (33.1%) men and 812 (66.9%) women, mean age 36.79 ± 12.50 years] completed the DeMask-20 instrument. Among them, 49.26% had an age lower than 35 years, 32.9% were between 35 to 49 years, while 17.8% were above 50 years. 326 (26.85%) were obliged to wear facemask during driving (professional drivers), while 730 (60.13%) had to wear mask at their working environment (professional workers). Regarding vulnerability to COVID-19, 11.8% of the participants were considered as high-risk group. Detailed demographic characteristics are presented in Table 3.

**Table 3:** Demographic characteristics.

Participants	N (%)	Age				Vulnerability	
		Mean ± SD	N (%)			N (%)	
			18–34 years	35–49 years	≥ 50 years	No	Yes
Total	1214	36.79 ± 12.50	598 (49.3)	400 (32.9)	216 (17.8)	1032 (88.2)	138 (11.8)
Male	402 (33.10)	40.43 ± 12.70	146 (36.3)	162 (40.3)	94 (23.4)	346 (87.8)	48 (12.2)
Female	812 (66.90)	34.99 ± 12.01	452 (55.7)	238 (29.3)	122 (15.0)	686 (88.4)	90 (11.6)
Professional drivers	326 (26.85)	37.01 ± 11.66	156 (47.9)	124 (38.0)	46 (14.1)	268 (85.9)	44 (14.1)
Professional workers	730 (60.13)	37.53 ± 11.41	320 (43.8)	280 (38.4)	130 (17.8)	610 (87.4)	88 (12.6)

N: Number of participants; SD: Standard Deviation.

36.7% of study participants used spectacles for distance activities, 12.4% for near activities, while 10.7% both for distance and near activities. 77.1% had never used contact lenses, 6.9% used them rarely, 6.9% frequently and 9.1% in a daily basis. 39.5% of the participants had a reported bDVA of 20/20, 11.4%, 4.3%, 3.0%, and 2.1% had a bDVA of 20/25, 20/50–20/32, 20/200–20/63, and < 20/200, respectively. 39.7% provided no information regarding their bDVA. Detailed clinical characteristics of the participants are presented in Tables 4 and 5.

**Table 4:** Spectacle and contact lens use.

Participants	Spectacles				Contact lenses			
	Ν (%)				Ν (%)			
	No use	For distance	For near	For distance and near	No use	Rarely	Frequently	Almost always
Total	488 (40.2)	446 (36.7)	150 (12.4)	130 (10.7)	936 (77.1)	84 (6.9)	84 (6.9)	110 (9.1)
Male	182 (45.3)	112 (27.9)	56 (13.9)	52 (12.9)	340 (84.6)	14 (3.5)	28 (6.9)	20 (5.0)
Female	306 (37.7)	334 (41.1)	94 (11.6)	78 (9.6)	596 (73.4)	70 (8.6)	56 (6.9)	90 (11.1)
Professional drivers	128 (39.3)	118 (36.2)	40 (12.3)	40 (12.3)	252 (77.3)	18 (5.5)	28 (8.6)	28 (8.6)
Professional workers	278 (38.1)	264 (36.1)	94 (12.9)	94 (12.9)	564 (77.2)	40 (5.5)	54 (7.4)	72 (9.9)

N: Number of participants.

**Table 5:** Reported binocular distance visual acuity.

Participants	Reported binocular Distance Visual Acuity					
	N (%)					
	No information	< 20/200	20/200–20/63	20/50–20/32	20/25	20/20
Total	482 (39.7)	26 (2.1)	36 (3.0)	52 (4.3)	138 (11.4)	480 (39.5)
Male	140 (34.8)	6 (1.5)	12 (3.0)	14 (3.5)	38 (9.4)	192 (47.8)
Female	342 (42.1)	20 (2.5)	24 (3.0)	38 (4.7)	100 (12.3)	288 (35.4)
Professional drivers	124 (38.0)	16 (4.9)	14 (4.3)	24 (7.4)	30 (9.2)	118 (36.2)
Professional workers	164 (26.0)	20 (3.2)	34 (5.4)	40 (6.3)	70 (11.1)	302 (48.0)

N: Number of participants.

**Fig 2:**
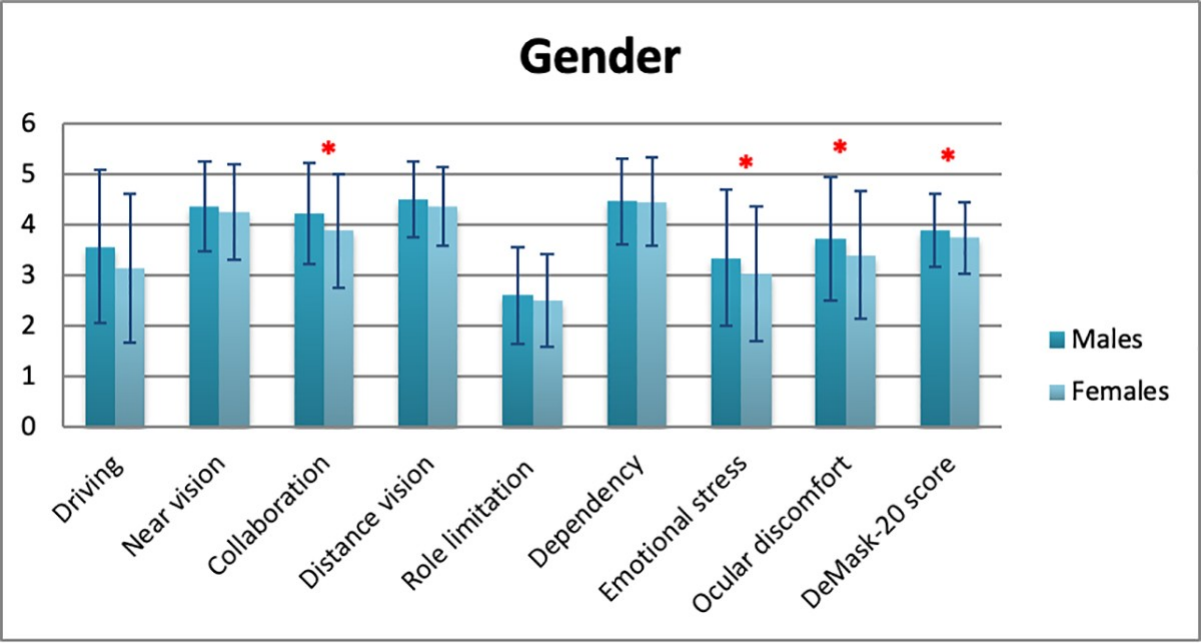
DeMask-20 score and subscores according to gender. *statistical significance, error bars: standard deviation.

**Fig 3:**
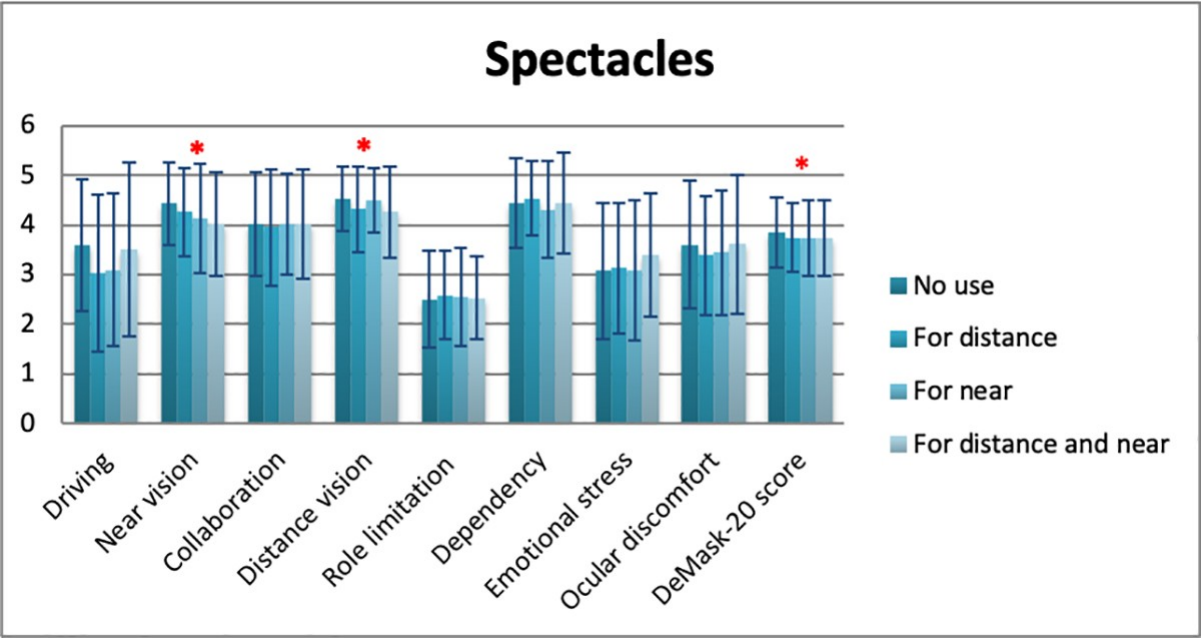
DeMask-20 score and subscores according to spectacle use. *statistical significance, error bars: standard deviation.

**Fig 4:**
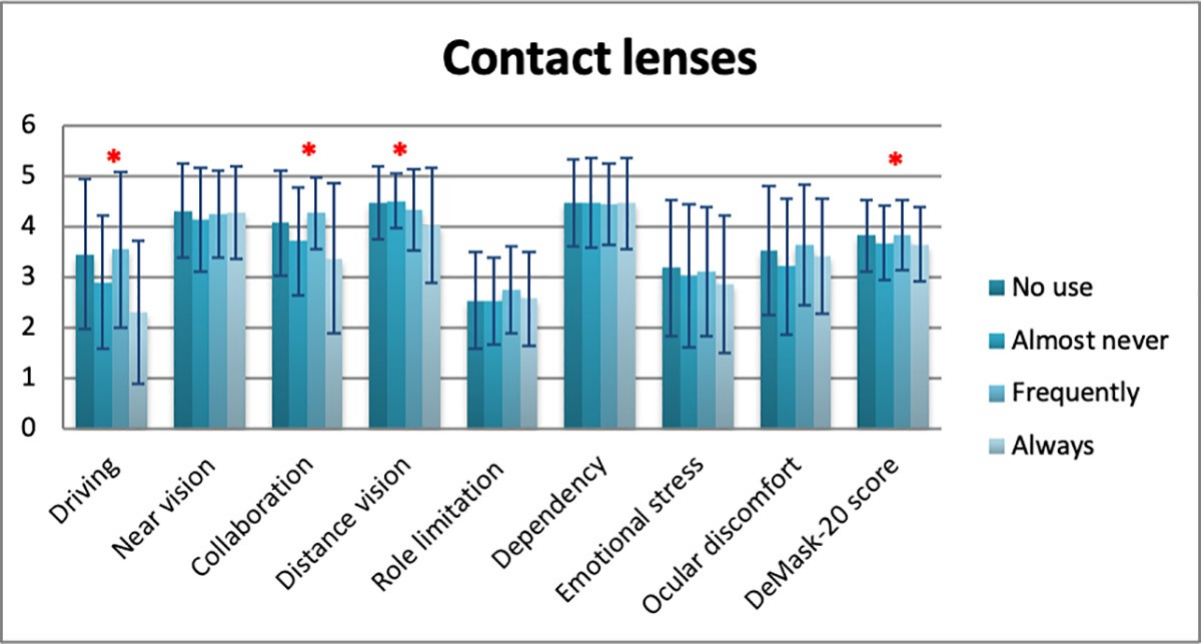
DeMask-20 score and subscores according to contact lens use. *statistical significance, error bars: standard deviation.

**Fig 5:**
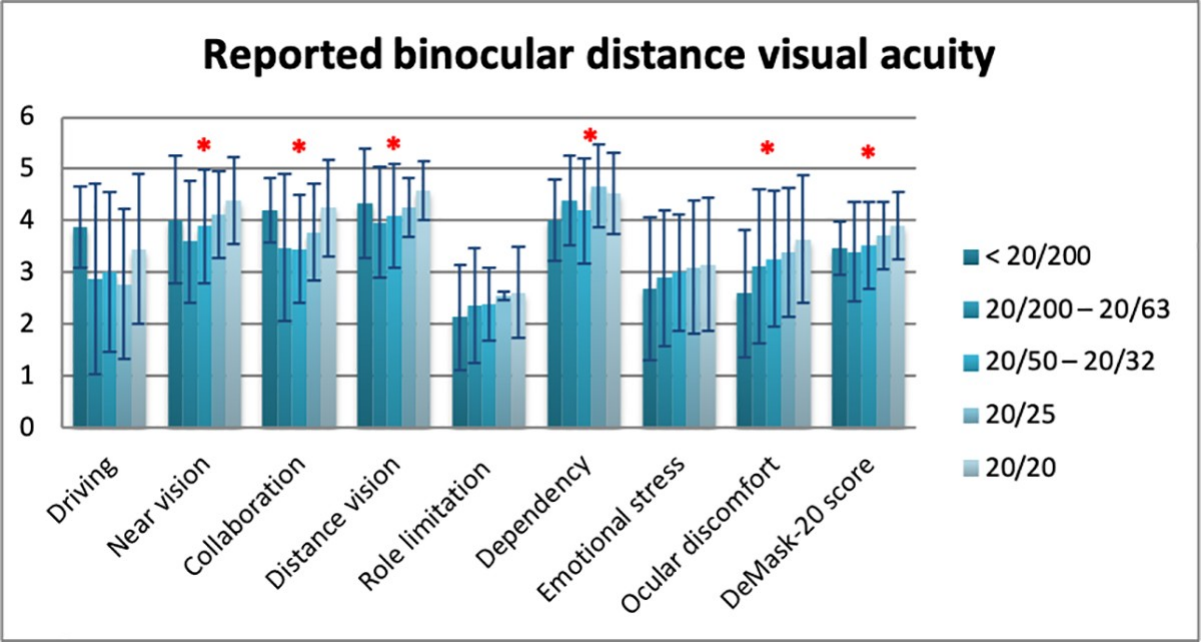
DeMask-20 score and subscores according to reported binocular distance visual acuity. *statistical significance, error bars: standard deviation.

**Fig 6:**
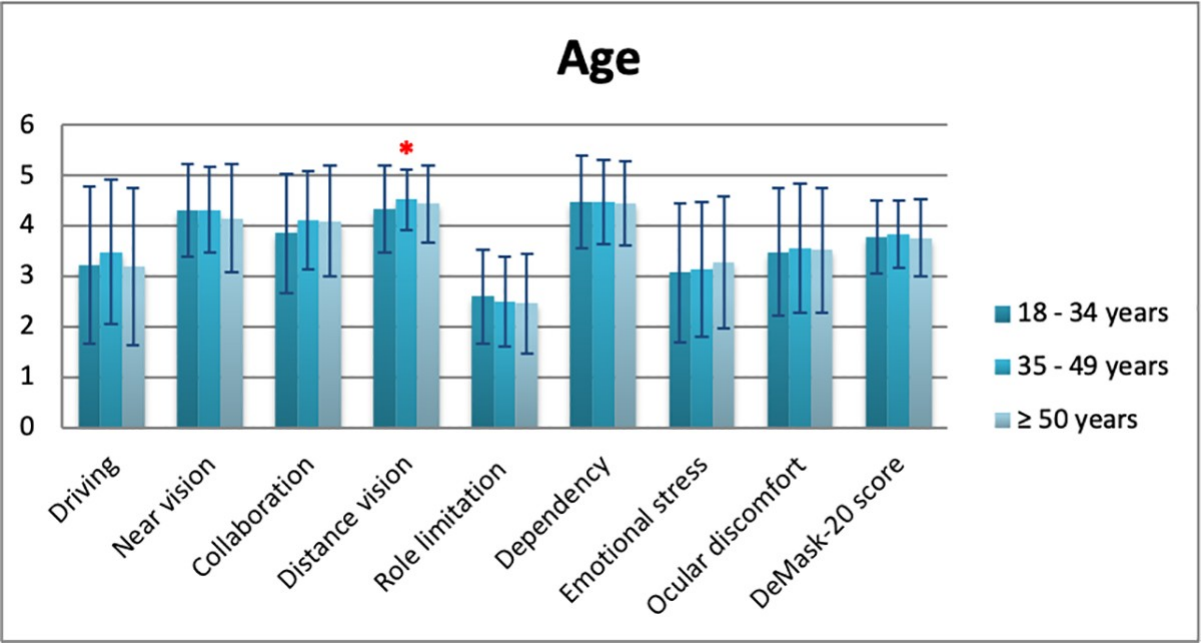
DeMask-20 score and subscores according to age. *statistical significance, error bars: standard deviation.

**Fig 7:**
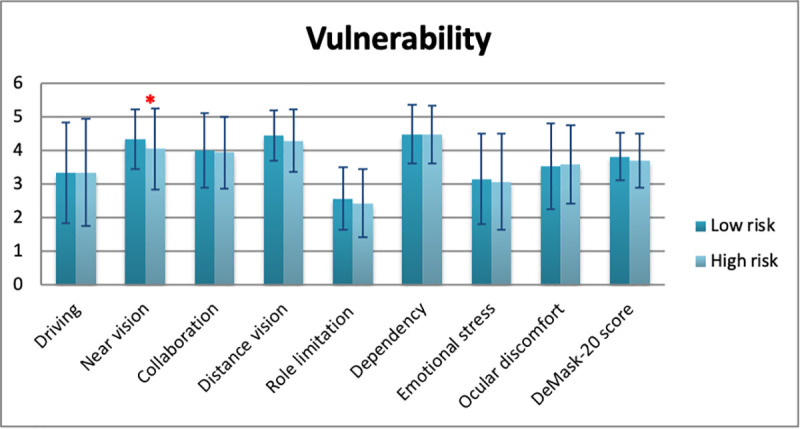
DeMask-20 score and subscores according to vulnerability. *statistical significance, error bars: standard deviation.

Mean DeMask-20 score of all study participants was 3.79 ± 0.71 (5 = no difficulty/discomfort, 1 = significant difficulty/discomfort) (Table 6). Significant differences in DeMask-20 score were detected in gender (men: 3.90 ± 0.72, women: 3.74 ± 0.70, p = 0.009), spectacle use (p = 0.034), contact lens use (p = 0.049), and bDVA (p = 0.001) (Tables 7–10, Figs 2–5), while no significant differences in DeMask-20 score were found in age (p = 0.751) and vulnerability (p = 0.199) (Tables 11 and 12, Figs 6 and 7). Regarding subscale scores, women demonstrated worse scores in collaboration [3.88 ± 1.12 vs 4.23 ± 1.00 (men), p = 0.003], emotional stress [3.03 ± 1.34 vs 3.34 ± 1.35 (men), p = 0.007], and ocular discomfort [3.40 ± 1.27 vs 3.73 ± 1.23 (men), p = 0.003] (Table 7). Facemask and spectacle use were associated with more difficulty in distance vision subscale (p = 0.008), and near vision subscale (p = 0.002) (Table 8). On the other hand, facemask and contact lens use were associated with more difficulty in driving (p = 0.037), collaboration (p = 0.001) and distance vision subscales (p = 0.001) (Table 9). Finally, facemask and bDVA were associated with more difficulty in distance vision subscale (p < 0.001) and near vision subscale (p = 0.001), collaboration (p < 0.001), dependency on others (p = 0.013), and ocular discomfort (p = 0.012) (Table 10).

**Table 6:** Overall and subscale scores of DeMask-20 instrument for the total number of participants.

Subscales	Total
	Mean ± SD [95% CI]
Driving	3.32 ± 1.51 [3.16–3.48]
Near vision	4.29 ± 0.93 [4.21–4.36]
Collaboration	3.99 ± 1.09 [3.91–4.07]
Distance vision	4.42 ± 0.77 [4.35–4.48]
Role limitation	2.54 ± 0.94 [2.47–2.62]
Dependency	4.46 ± 0.87 [4.39–4.53]
Emotional stress	3.13 ± 1.35 [3.03–3.24]
Ocular discomfort	3.51 ± 1.27 [3.41–3.61]
**DeMask-20 score**	3.79 ± 0.71 [3.75–3.83]

CI: Confidence Interval; SD: Standard Deviation.

**Table 7:** Overall and subscale scores of DeMask-20 instrument according to gender.

Subscales	Gender		
	Mean ± SD [95% CI]		p value
	Males	Females	
Driving	3.57 ± 1.52 [3.40–3.73]	3.14 ± 1.48 [2.98–3.30]	0.074
Near vision	4.37 ± 0.89 [4.25–4.49]	4.25 ± 0.95 [4.15–4.34]	0.128
Collaboration	4.23 ± 1.00 [4.16–4.30]	3.88 ± 1.12 [3.80–3.96]	0.003*
Distance vision	4.50 ± 0.75 [4.39–4.60]	4.37 ± 0.78 [4.30–4.45]	0.064
Role limitation	2.61 ± 0.96 [2.48–2.74]	2.51 ± 0.92 [2.42–2.60]	0.222
Dependency	4.47 ± 0.85 [4.35–4.59]	4.46 ± 0.88 [4.37–4.54]	0.899
Emotional stress	3.34 ± 1.35 [3.16–3.53]	3.03 ± 1.34 [2.90–3.16]	0.007*
Ocular discomfort	3.73 ± 1.23 [3.56–3.90]	3.40 ± 1.27 [3.27–3.52]	0.003*
**DeMask-20 score**	3. 90 ± 0.72 [3.86–3.94]	3.74 ± 0.70 [3.70–3.78]	0.009*

*p < 0.01; CI: Confidence Interval; SD: Standard Deviation.

**Table 8:** Overall and subscale scores of DeMask-20 instrument according to spectacle use.

Subscales	Spectacles				
	Mean ± SD [95% CI]				p value
	No use	For distance	For near	For distance and near	
Driving	3.59 ± 1.32 [3.45–3.73]	3.03 ± 1.58 [2.86–3.2]	3.10 ± 1.54 [2.93–3.27]	3.50 ± 1.75 [3.31–3.69]	0.177
Near vision	4.43 ± 0.84 [4.38–4.48]	4.26 ± 0.90 [4.21–4.31]	4.13 ± 1.10 [4.07–4.19]	4.01 ± 1.05 [3.95–4.07]	0.002*
Collaboration	4.02 ± 1.05 [3.94–4.10]	3.95 ± 1.17 [3.87–4.03]	4.02 ± 1.01 [3.95–4.09]	4.02 ± 1.09 [3.94–4.10]	0.943
Distance vision	4.52 ± 0.65 [4.48–4.56]	4.32 ± 0.86 [4.27–4.37]	4.50 ± 0.65 [4.46–4.54]	4.26 ± 0.91 [4.21–4.31]	0.008*
Role limitation	2.50 ± 0.98 [4.44–4.56]	2.59 ± 0.90 [2.54–2.64]	2.56 ± 0.99 [2.50–2.62]	2.53 ± 0.83 [2.48–2.58]	0.772
Dependency	4.45 ± 0.91 [4.40–4.50]	4.54 ± 0.75 [4.50–4.58]	4.31 ± 0.97 [4.26–4.36]	4.44 ± 1.01 [4.38–4.50]	0.251
Emotional stress	3.08 ± 1.37 [3.00–3.16]	3.13 ± 1.32 [3.06–3.20]	3.08 ± 1.42 [3.00–3.16]	3.40 ± 1.25 [3.33–3.47]	0.390
Ocular discomfort	3.60 ± 1.29 [3.53–3.67]	3.39 ± 1.20 [3.32–3.46]	3.44 ± 1.25 [3.37–3.51]	3.61 ± 1.39 [3.53–3.69]	0.278
**DeMask-20 score**	3.85 ± 0.70 [3.81–3.89]	3.75 ± 0.69 [3.71–3.79]	3.73 ± 0.76 [3.69–3.77]	3.73 ± 0.77 [3.69–3.77]	0.034**

*p < 0.01

** p < 0.05; CI: Confidence Interval; SD: Standard Deviation.

**Table 9:** Overall and subscale scores of DeMask-20 instrument according to contact lens use.

Subscales	Contact lenses				
	Mean ± SD [95% CI]				p value
	No use	Almost never	Frequently	Always	
Driving	3.44 ± 1.49 [3.28–3.60]	2.89 ± 1.32 [2.75–3.03]	3.54 ± 1.54 [3.37–3.71]	2.29 ± 1.41 [2.14–2.44]	0.037**
Near vision	4.31 ± 0.93 [4.26–4.36]	4.13 ± 1.02 [4.07–4.19]	4.24 ± 0.85 [4.19–4.29]	4.26 ± 0.92 [4.21–4.31]	0.643
Collaboration	4.07 ± 1.04 [3.99–4.15]	3.70 ± 1.08 [3.62–3.78]	4.26 ± 0.71 [4.21–4.31]	3.36 ± 1.48 [4.26–0.71]	0.001*
Distance vision	4.46 ± 0.72 [4.42–4.50]	4.50 ± 0.55 [4.47–4.53]	4.33 ± 0.81 [4.28–4.38]	4.03 ± 1.14 [3.97–4.09]	0.001*
Role limitation	2.53 ± 0.95 [2.48–2.58]	2.52 ± 0.87 [2.47–2.57]	2.74 ± 0.87 [2.69–2.79]	2.56 ± 0.94 [2.51–2.61]	0.569
Dependency	4.46 ± 0.87 [2.41–2.51]	4.46 ± 0.89 [2.41–2.51]	4.43 ± 0.81 [2.38–2.48]	4.45 ± 0.91 [2.40–2.50]	0.995
Emotional stress	3.18 ± 1.35 [3.10–3.26]	3.02 ± 1.42 [2.94–3.10]	3.10 ± 1.28 [3.03–3.17]	2.84 ± 1.36 [2.76–2.92]	0.311
Ocular discomfort	3.53 ± 1.28 [3.46–3.6]	3.20 ± 1.35 [3.12–3.28]	3.63 ± 1.20 [3.56–3.70]	3.41 ± 1.13 [3.35–3.47]	0.324
**DeMask-20 score**	3.81 ± 0.71 [3.77–3.85]	3.67 ± 0.73 [3.63–3.71]	3.83 ± 0.69 [3.79–3.87]	3.64 ± 0.74 [3.60–3.68]	0.049**

*p < 0.01

** p < 0.05; CI: Confidence Interval; SD: Standard Deviation.

**Table 10:** Overall and subscale scores of DeMask-20 instrument according to the reported binocular distance visual acuity.

Subscales	Reported binocular Distance Visual Acuity					
	Mean ± SD [95% CI]					p value
	< 20/200	20/200–20/63	20/50–20/32	20/25	20/20	
Driving	3.88 ± 0.79 [3.79–3.97]	2.86 ± 1.84 [2.66–3.06]	3.00 ± 1.55 [2.83–3.17]	2.77 ± 1.73 [2.58–2.96]	3.45 ± 1.45 [3.29–3.61]	0.321
Near vision	4.02 ± 1.23 [3.95–4.09]	3.60 ± 1.18 [3.53–3.67]	3.89 ± 1.10 [3.83–3.95]	4.12 ± 1.03 [4.06–4.18]	4.39 ± 0.84 [4.34–4.44]	0.001*
Collaboration	4.20 ± 0.63 [4.15–4.25]	3.47 ± 1.42 [3.37–3.57]	3.45 ± 1.05 [3.37–3.53]	3.77 ± 1.06 [3.69–3.85]	4.24 ± 0.93 [4.17–4.31]	< 0.001*
Distance vision	4.33 ± 1.05 [4.27–4.39]	3.96 ± 1.07 [3.90–4.02]	4.09 ± 0.99 [4.03–4.15]	4.26 ± 0.76 [4.22–4.30]	4.58 ± 0.57 [4.55–4.61]	< 0.001*
Role limitation	2.13 ± 1.01 [2.07–2.19]	2.35 ± 1.11 [2.29–2.41]	2.38 ± 0.71 [2.34–2.42]	2.55 ± 1.00 [2.49–2.61]	2.61 ± 0.88 [2.56–2.66]	0.243
Dependency	4.00 ± 0.79 [3.96–4.04]	4.39 ± 0.87 [4.34–4.44]	4.19 ± 1.01 [4.13–4.25]	4.67 ± 0.65 [4.63–4.71]	4.53 ± 0.79 [4.49–4.57]	0.013**
Emotional stress	2.69 ± 1.38 [2.61–2.77]	2.89 ± 1.32 [2.82–2.96]	3.00 ± 1.12 [2.94–3.06]	3.09 ± 1.38 [3.01–3.17]	3.15 ± 1.29 [3.08–3.22]	0.691
Ocular discomfort	2.59 ± 1.23 [2.52–2.66]	3.11 ± 1.50 [3.03–3.19]	3.26 ± 1.31 [3.19–3.33]	3.39 ± 1.19 [3.32–3.46]	3.64 ± 1.24 [3.57–3.71]	0.012**
**DeMask-20 score**	3.46 ± 0.51 [3.43–3.49]	3.39 ± 0.96 [3.34–3.44]	3.52 ± 0.85 [3.47–3.57]	3.70 ± 0.71 [3.66–3.74]	3.90 ± 0.65 [3.86–3.94]	0.001*

*p < 0.01

** p < 0.05; CI: Confidence Interval; SD: Standard Deviation.

**Table 11:** Overall and subscale scores of DeMask-20 instrument according to age.

Subscales	Age			
	Mean ± SD [95% CI]			p value
	18–34 years	35–49 years	≥ 50 years	
Driving	3.23 ± 1.56 [3.06–3.4]	3.48 ± 1.44 [3.32–3.64]	3.20 ± 1.56 [3.03–3.37]	0.583
Near vision	4.32 ± 0.92 [4.27–4.37]	4.32 ± 0.86 [4.27–4.37]	4.15 ± 1.07 [4.09–4.21]	0.217
Collaboration	3.85 ± 1.18 [3.76–3.94]	4.11 ± 0.97 [4.04–4.18]	4.09 ± 1.1 [4.01–4.17]	0.082
Distance vision	4.33 ± 0.87 [4.28–4.38]	4.52 ± 0.59 [4.49–4.55]	4.44 ± 0.76 [4.40–4.48]	0.024**
Role limitation	2.60 ± 0.94 [2.55–2.65]	2.50 ± 0.90 [2.45–2.55]	2.46 ± 0.99 [2.4–2.52]	0.282
Dependency	4.47 ± 0.92 [4.42–4.52]	4.47 ± 0.83 [4.42–4.52]	4.44 ± 0.83 [4.39–4.49]	0.942
Emotional stress	3.08 ± 1.38 [3.00–3.16]	3.14 ± 1.33 [3.07–3.21]	3.28 ± 1.30 [3.21–3.35]	0.428
Ocular discomfort	3.48 ± 1.27 [3.41–3.55]	3.55 ± 1.28 [3.48–3.62]	3.52 ± 1.24 [3.45–3.59]	0.840
**DeMask-20 score**	3.77 ± 0.72 [3.73–3.81]	3.83 ± 0.66 [3.79–3.87]	3.76 ± 0.77 [3.72–3.80]	0.751

** p < 0.05; CI: Confidence Interval; SD: Standard Deviation.

**Table 12:** Overall and subscale scores of DeMask-20 instrument according to vulnerability.

Subscales	Vulnerability		
	Mean ± SD [95% CI]		p value
	Low risk	High risk	
Driving	3.32 ± 1.51 [3.16–3.48]	3.33 ± 1.60 [3.16–3.5]	0.990
Near vision	4.32 ± 0.89 [4.24–4.39]	4.04 ± 1.21 [3.75–4.33]	0.021**
Collaboration	4.00 ± 1.11 [3.92–4.08]	3.93 ± 1.07 [3.85–4.01]	0.716
Distance vision	4.43 ± 0.75 [4.37–4.50]	4.28 ± 0.93 [4.06–4.50]	0.130
Role limitation	2.55 ± 0.93	2.42 ± 1.02	0.276
Dependency	4.47 ± 0.88 [4.40–4.55]	4.46 ± 0.87 [4.39–4.53]	0.839
Emotional stress	3.14 ± 1.35 [3.03–3.26]	3.06 ± 1.42 [2.72–3.40]	0.624
Ocular discomfort	3.52 ± 1.28 [3.41–3.63]	3.58 ± 1.17 [3.30–3.87]	0.689
**DeMask-20 score**	3.80 ± 0.71 [3.76–3.84]	3.69 ± 0.81 [3.64–3.74]	0.199

** p < 0.05; CI: Confidence Interval; SD: Standard Deviation.

Mean compliance of all participants was 4.05 ± 0.96 (best = 5, worst = 1). Differences in compliance were detected in gender [3.92 ± 1.70 (men), 4.11 ± 0.90 (women), p = 0.028], age [4.02 ± 0.97 (< 50 years), 4.26 ± 0.80 (≥ 50 years), p = 0.014], and spectacle use [3.91 ± 1.04 (spectacles), 4.14 ± 0.89 (no spectacles), p = 0.004]. Moreover, professional workers and professional drivers demonstrated significantly better compliance (p = 0.008 and p = 0.047). All compliance scores are presented in Table 13.

**Table 13:** Compliance.

Compliance scores	Mean ± SD [95% CI]				p value
Gender	*Men*	3.92 ± 1.07 [3.81–4.03]	*Women*	4.11 ± 0.90 [4.05–4.17]	0.028**
Age	*< 50 years*	4.02 ± 0.97 [3.96–4.08]	*≥ 50 years*	4.26 ± 0.80 [4.15–4.37]	0.014**
Spectacles	*Yes*	3.91 ± 1.04 [3.83–3.99]	*No*	4.14 ± 0.89 [4.06–4.22]	0.004*
Contact lenses	*Yes*	4.06 ± 0.93 [3.95–4.17]	*No*	4.04 ± 0.97 [3.98–4.10]	0.830
bDVA	*20/20*	4.14 ± 0.81 [4.07–4.21]	*< 20/200–20/25*	4.04 ± 0.94 [3.96–4.12]	0.306
Working status	*Workers*	4.13 ± 0.89 [4.07–4.19]	*Non-workers*	3.92 ± 1.05 [3.83–4.01]	0.008*
Driving	*Drivers*	4.17 ± 0.82 [4.08–4.26]	*Non-drivers*	4.00 ± 1.01 [3.93–4.07]	0.047**
Vulnerability	*Low risk*	4.05 ± 0.95 [3.99–4.11]	*High risk*	4.00 ± 1.06 [3.85–4.15]	0.660

*p < 0.01

** p < 0.05; bDVA: binocular distance visual acuity; CI: Confidence Interval; SD: Standard Deviation.

Significant correlation was detected between compliance and DeMask-20 score (p < 0.001, R^2^ = 0.471). Correlations of compliance with subscale scores are presented in Table 14. Significant correlations were detected with driving (p = 0.005, R^2^ = 0.467), near vision (p < 0.001, R^2^ = 0.493), distance vision (p < 0.001, R^2^ = 0.386), collaboration (p < 0.001, R^2^ = 0.492), role limitation (p < 0.001, R^2^ = 0.443), emotional stress (p < 0.001, R^2^ = 0.411).

**Table 14:** Scores and correlations of DeMask-20 subscales and DeMask-20 instrument with compliance to facemask-wearing directive.

Subscales	Compliance to facemask-wearing directive						
	Mean ± SD [95% CI]					R^2^	p value
	Yes, always	Yes, almost always	Sometimes	No, almost never	No, never		
Driving	3.69 ± 1.37 [3.54–3.84]	3.25 ± 1.54 [3.08–3.42]	2.68 ± 1.52 [2.52–2.85]	3 (SD: NA)	3.00 ± 2.83 [2.69–3.31]	0.467	0.0049*
Near vision	4.50 ± 0.71 [4.46–4.54]	4.28 ± 0.86 [4.23–4.33]	4.06 ± 1.12 [4.00–4.12]	4.12 ± 1.02 [4.06–4.18]	3.4 ± 1.61 [3.31–3.49]	0.493	< 0.0001*
Collaboration	4.29 ± 0.89 [4.23–4.35]	3.94 ± 1.02 [3.87–4.01]	3.53 ± 1.34 [3.43–3.63]	3.25 ± 1.71 [3.13–3.37]	3.56 ± 1.94 [3.42–3.70]	0.492	< 0.0001*
Distance vision	4.56 ± 0.58 [4.53–4.59]	4.38 ± 0.79 [4.34–4.42]	4.28 ± 0.90 [4.23–4.33]	4.43 ± 0.98 [4.37–4.49]	4.08 ± 1.19 [4.01–4.15]	0.386	0.0002*
Role limitation	2.79 ± 0.99 [2.73–2.85]	2.46 ± 0.84 [2.41–2.51]	2.34 ± 0.93 [2.29–2.39]	2.10 ± 0.94 [2.05–2.15]	2.21 ± 0.99 [2.15–2.27]	0.443	< 0.0001*
Dependency	4.49 ± 0.91 [4.44–4.54]	4.51 ± 0.81 [4.46–4.56]	4.27 ± 0.94 [4.22–4.32]	4.30 ± 0.95 [4.25–4.35]	4.67 ± 0.66 [4.63–4.71]	0.189	0.3792
Emotional stress	3.42 ± 1.33 [3.35–3.49]	3.14 ± 1.29 [3.07–3.21]	2.58 ± 1.27 [2.51–2.65]	2.70 ± 1.49 [2.62–2.78]	3.14 ± 1.65 [3.05–3.23]	0.411	< 0.0001*
Ocular discomfort	3.64 ± 1.31 [3.57–3.71]	3.50 ± 1.19 [3.43–3.57]	3.26 ± 1.27 [3.19–3.33]	3.27 ± 1.43 [3.19–3.35]	3.62 ± 1.47 [3.54–3.70]	0.268	0.0769
**DeMask-20 score**	3.97 ± 0.64 [3.93–4.01]	3.77 ± 0.65 [3.73–3.81]	3.54 ± 0.78 [3.50–3.58]	3.59 ± 0.95 [3.54–3.64]	3.48 ± 1.02 [3.42–3.54]	0.471	< 0.001*

*p: Correlation is significant at the 0.01 level (2-tailed); CI: Confidence Interval; NA: Not applicable (one participant); SD: Standard Deviation.

## Discussion

The COVID-19 pandemic has introduced the necessity of facemask use as a type of personal protection equipment (PPE) for the reduction of the SARS-CoV-2 transmission. A great variety of facemask types is available for the general public [9]. Among them, the traditional medical facemasks, known as “surgical masks”, some more specialized masks such as FFP2, FFP3, N95, KN95, but also homemade (cloth) non-certified facemasks have become part of daily life [9].

Despite the significant role that facemasks play during this pandemic due to the beneficial impact on the prevention of the virus SARS-CoV-2 transmission, they had traditionally been associated with discomfort and increased difficulty in certain activities of daily living [15, 26–29]. The perceived difficulty when wearing a facemask could easily contribute to reduced compliance to the facemask-wearing directive and potentially increase the rate of virus transmission. Moreover, former researchers reported that people who are using spectacles and/or contact lenses perceive significantly more difficulty when compared to the rest of the population, primarily due to fogging of glasses and intense tear evaporation, especially when the facemask is not properly fitted [15].

Within this context, we attempted to measure the perceived difficulty and/or discomfort of Greek people when wearing a facemask, and explore potential correlations with compliance to the facemask-wearing directive by the Ministry of Health and Welfare. Special attention was given to identify whether spectacle, contact lens use and suboptimal visual acuity contribute to lower levels of compliance.

Since no relevant validated instrument existed for Greek-speaking patients, we constructed the DeMask-20 questionnaire, which quantified the perceived difficulty when wearing a facemask in 20 items, grouped in 8 subscales. Factor analysis suggested that the DeMask-20 instrument demonstrates adequate validity, while Cronbach’s alpha indicated sufficient internal consistency for all subscales.

Our participants presented an average DeMask-20 score of 3.79 indicating that Greek people do actually perceive a variable amount of difficulty and discomfort when wearing a facemask. Women reported significantly worse scores than men, identifying difficulty in collaborating with peers, and due to ocular discomfort and emotional stress. Spectacles and contact lenses also contributed to worse DeMask-20 scores, primarily due to difficulty in distance and near vision activities (for spectacle users) and due to distance vision activities, collaboration and driving (for contact lens users). Moreover, lower levels of visual acuity were associated with worse DeMask-20 scores.

Despite the different methods, our outcomes are in accordance to former publications, which also revealed a negative impact on the quality of life when using a facemask. Morishima et al. [15] performed a repeated cross-sectional survey in Japan in 2009, 2012 and 2015 for the use of facemask for the protection from H1N1 and common cold viruses. According to their outcomes, the most common problem was humidity in the facemask, fogging up of glasses, difficulty in breathing for both genders, and makeup coming off for women. Similarly, Lim et al. [26] and Ong et al. [14] analyzed the impact of PPE such as N95 facemask on the development of headaches of healthcare workers while attending to patients during the 2003 SARS epidemic and COVID-2019 pandemic, respectively. From these surveys, it was concluded that de novo PPE-associated headaches or exacerbation of pre-existing headache disorders are developed in the majority of healthcare workers, which leads to frequent abuse of analgesics. Another phenomenon described in the literature during COVID-19 pandemic is retroauricular mask-induced dermatitis, which is caused by ear loop facemasks [13].

Ocular problems due to facemask use were reported by Moshirfar et al. [27] who indicated that, during the COVID-19 pandemic, an increase in ocular irritation and dry eye disease symptoms was observed among people using a facemask regularly, patients and healthcare workers. Among the possible explanations of this phenomenon is the tear film evaporation accelerated by the increased airflow toward the eyes, which may result in irritation or inflammation of the ocular surface when it lasts for hours or days. An additional interesting explanation about the corneal irritation among staff members using taped facemasks for the prevention of air convection toward the eyes is the fact that the adhesion of the tape to the skin of the upper cheek may prevent the lower eyelid from normal excursion resulting in mechanical ectropion with secondary lagophthalmos. In fact, the same authors hypothesized that dry eye caused by evaporating of the tear film, an essential barrier against pathogenic invasion, but simultaneously the increase of eye rubbing because of the ocular discomfort could result in a higher vulnerability to pathogens through the eyes.

Regarding compliance to the facemask-wearing directive, 76% of study participants declared full or almost full compliance, 18.1% sometimes, and 5.1% no compliance. Women complied more than men despite worse DeMask-20 scores, age was positively correlated with compliance, and professionals who were obliged to wear a facemask in their working environment (including professional drivers) presented better compliance, as well. As expected, spectacles were associated with significantly worse compliance.

Compliance was significantly correlated with the total DeMask-20 score and almost all subscale scores. This outcome provides essential new information for some of the fundamental reasons that explain why a person complies with the facemask directive, or not. Within this context, it provides the necessary data to the Healthcare authorities to implement strategies or interventions to improve compliance rates. Therefore, the primary target group for the Greek Ministry of Health and Welfare should be males, below 50 years old, who wear spectacles. Secondary, they should focus on people who: a) have average or poor visual acuity and experience significant difficulty both in near and distance vision activities, and, b) who have a pre-existing ocular surface disease that is most likely to be exacerbated by the facemask. Poor compliance is most likely also on people who present difficulty when collaborating with others, and those who experience significant emotional stress. Last but not least, poor compliance is expected in people who believe that facemask use reduces their opportunities for personal growth.

Former investigators reported similar results regarding compliance to the facemask-wearing directive. Sim et al. indicated personal discomfort and sense of embarrassment as the primary reasons for reduced compliance [30]. Regarding healthcare providers primary reasons for reduced compliance were discomfort, breathing problems and shortness of breath [31–33]. Moreover, young age and male gender were associated with reduced compliance [34]. In fact, according to Capraro &Barcelo, men presented significantly lower rates of compliance than women, especially when facemasks were not obligatory by law. Interestingly, it has also been found that focusing on community protection was associated with higher compliance to the facemask directive than focusing on protecting the individuals themselves [35].

Prior to the interpretation of our results, certain limitations of our study have to be noted. Although we have a robust number of participants, our sample was not stratified. Moreover, since participants were contacted via Facebook, only patients who use Social Media were represented. However, taking into account the significant penetrance of the internet and the social media to the Greek society, we are confident that our outcomes could be generalized for Greece.

## Conclusions

To our knowledge, this is the first study that assesses compliance to the facemask-wearing directive in Greece. Moreover, to our knowledge, this is the first study to construct a validated instrument that evaluates the perceived difficulty when wearing a facemask. Within this context, we provided the necessary methods that could evaluate the compliance trends and the efficacy of healthcare interventions in the COVID-19 era. Nevertheless, our outcomes suggest that young males who use spectacles should be targeted by Greek Healthcare authorities in order to improve compliance rates. Further studies with larger stratified cohorts are necessary to confirm our results and contribute to the body of knowledge of this important subject.

## Supporting information

S1 File DeMask-20 Questionnaire in Greek. (PDF)

S2 File DeMask-20 Questionnaire in English. (PDF)
