# Supplementary material for: Determinants of compliance to the facemask directive in Greece: A population study
Source: PLoS One. 2021 Mar 19;16(3):e0248929. doi: 10.1371/journal.pone.0248929 (PMC7978275; doi:10.1371/journal.pone.0248929)
Supplement: S1 File — (PDF) [file pone.0248929.s001.pdf]

DeMask-20 - Ερωτηματολόγιο αξιολόγησης ποιότητας ζωής για τη χρήση μάσκας προσώπου

Οφθαλμολογική Κλινική, Πανεπιστημιακό Γενικό Νοσοκομείο Αλεξανδρούπολης
Δημοκρίτειο Πανεπιστήμιο Θράκης

<u>Μέρος Α</u>
Αρχικά Ονόματος: _________

Φύλο

a. Άντρας
b. Γυναίκα

Έτος Γέννησης: ________

Χρησιμοποιείτε γυαλιά;

a. Όχι
b. Ναι για μακριά
c. Ναι για κοντά
d. Ναι για μακριά και κοντά

Χρησιμοποιείτε φακούς επαφής;

a. Όχι
b. Ναι, σπάνια
c. Ναι, συχνά
d. Ναι, σχεδόν πάντα

Ανήκετε σε ευπαθή ομάδα*;

a. Όχι
b. Ναι
c. Δεν ξέρω

***Ευπαθής Ομάδα θεωρείται σύμφωνα με το ΦΕΚ 1856/15-05-2020**
Ηλικία άνω των 65 ετών / Βαριές καρδιοπάθειες / Βαριές Πνευμονοπάθειες / Ανθεκτική αρτηριακή υπέρταση / Αρρύθμιστος σακχαρώδης διαβήτης / Βαριές νευρολογικές / νευρομυϊκές παθήσεις / Νεφρική ανεπάρκεια / Υψηλός δείκτης μάζας σώματος / Νεοπλασίες / Ανοσοκαταστολή / Εγκυμοσύνη

Γνωρίζετε τη μακρινή σας οπτική οξύτητα (και στα δύο μάτια); Παρακαλώ σημειώστε την όραση σας με διόρθωση (δηλαδή με γυαλιά ή φακούς επαφής αν φοράτε).

a. Όχι
b. Κάτω από 1/10
c. Μεταξύ 1/10 και 3/10
d. Μεταξύ 4/10 και 6/10
e. 7/10 ή 8/10
f. Σχεδόν άριστη (9/10 ή 10/10)

Συμμορφώνεστε με τις οδηγίες του Υπουργείου Υγείας για τη χρήση μάσκας προσώπου;

a. Όχι
b. Μάλλον όχι
c. Σχετικά (μερικές φορές)
d. Ναι, τις περισσότερες φορές
e. Ναι, απόλυτα

Οι ερωτήσεις 1 & 2 αφορούν άτομα που επιβάλλεται να φορούν μάσκα κατά τη διάρκεια της οδήγησης (πχ. οδηγοί μέσων μαζικής μεταφοράς) ή φορούν μάσκα κατά την οδήγηση για δικούς τους λόγους (πχ. ανήκουν σε ευπαθείς ομάδες). Στην περίπτωση που δεν ανήκετε σε αυτές τις κατηγορίες παρακαλώ προχωρήστε στην ερώτηση 3.

1. Όταν φοράτε μάσκα, πόσο δυσκολεύεστε όταν οδηγείτε μέρα σε περιοχές που γνωρίζετε καλά;

a. Αναγκάζομαι να βγάζω τη μάσκα σε αυτή την περίπτωση
b. Σχεδόν έχω σταματήσει να οδηγώ λόγω της όρασής μου και της χρήσης μάσκας
c. Δυσκολεύομαι πολύ
d. Δυσκολεύομαι αρκετά
e. Δυσκολεύομαι πολύ λίγο
f. Δεν έχω καμία δυσκολία

2. Όταν φοράτε μάσκα, πόσο δυσκολεύεστε όταν οδηγείτε νύχτα, με δύσκολες συνθήκες ή σε περιοχές που δε γνωρίζετε;

a. Αναγκάζομαι να βγάζω τη μάσκα σε αυτή την περίπτωση
b. Σχεδόν έχω σταματήσει να οδηγώ νύχτα ή σε δύσκολες συνθήκες ή σε περιοχές που δε γνωρίζω λόγω της όρασής μου και της χρήσης μάσκας
c. Δυσκολεύομαι πολύ
d. Δυσκολεύομαι αρκετά
e. Δυσκολεύομαι πολύ λίγο
f. Δεν έχω καμία δυσκολία

Οι ερωτήσεις 3, 4 & 5 αφορούν άτομα που επιβάλλεται να φορούν μάσκα κατά τη διάρκεια της εργασίας τους (πχ. εργαζόμενοι σε νοσοκομεία ή στην εστίαση) ή φορούν μάσκα στο χώρο εργασίας για δικούς τους λόγους (πχ. ανήκουν σε ευπαθείς ομάδες). Στην περίπτωση που δεν ανήκετε σε αυτές τις κατηγορίες παρακαλώ προχωρήστε στην ερώτηση 6.

3. Όταν φοράτε μάσκα, πόσο δυσκολεύεστε στην εργασία σας να διαβάσετε ένα έντυπο υπηρεσιακό έγγραφο;

a. Αναγκάζομαι να βγάζω τη μάσκα σε αυτή την περίπτωση
b. Σχεδόν έχω σταματήσει να διαβάζω υπηρεσιακά έγγραφα λόγω της όρασής μου και της χρήσης μάσκας
c. Δυσκολεύομαι πολύ
d. Δυσκολεύομαι αρκετά
e. Δυσκολεύομαι πολύ λίγο
f. Δεν έχω καμία δυσκολία

4. Όταν φοράτε μάσκα, πόσο δυσκολεύεστε στην εργασία σας να διαβάσετε ένα κείμενο στον υπολογιστή;

a. Αναγκάζομαι να βγάζω τη μάσκα σε αυτή την περίπτωση
b. Σχεδόν έχω σταματήσει να διαβάζω κείμενο στον υπολογιστή λόγω της όρασής μου και της χρήσης μάσκας
c. Δυσκολεύομαι πολύ
d. Δυσκολεύομαι αρκετά
e. Δυσκολεύομαι πολύ λίγο
f. Δεν έχω καμία δυσκολία

5. Όταν φοράτε μάσκα, πόσο δυσκολεύεστε στην εργασία σας να συνεργαστείτε με ένα συνάδελφο;

a. Αναγκάζομαι να βγάζω τη μάσκα σε αυτή την περίπτωση
b. Σχεδόν έχω σταματήσει να συνεργάζομαι με συναδέλφους λόγω της όρασής μου και της χρήσης μάσκας
c. Δυσκολεύομαι πολύ
d. Δυσκολεύομαι αρκετά
e. Δυσκολεύομαι πολύ λίγο
f. Δεν έχω καμία δυσκολία

Οι επόμενες ερωτήσεις αφορούν όλους

6. Όταν φοράτε μάσκα πόσο δυσκολεύεστε να αναγνωρίσετε γνωστούς σας ανθρώπους που βρίσκονται στο απέναντι πεζοδρόμιο;

a. Αναγκάζομαι να βγάζω τη μάσκα σε αυτή την περίπτωση
b. Σχεδόν δεν αναγνωρίζω γνωστούς ανθρώπους λόγω της όρασης μου και της χρήσης μάσκας
c. Δυσκολεύομαι πολύ
d. Δυσκολεύομαι αρκετά
e. Δυσκολεύομαι πολύ λίγο
f. Δεν έχω καμία δυσκολία

7. Όταν φοράτε μάσκα πόσο δυσκολεύεστε να διαβάζετε πινακίδες δρόμων ή καταστημάτων;

a. Αναγκάζομαι να βγάζω τη μάσκα σε αυτή την περίπτωση
b. Σχεδόν δεν μπορώ να διαβάσω πινακίδες δρόμων ή καταστημάτων λόγω της όρασής μου και της χρήσης μάσκας
c. Δυσκολεύομαι πολύ
d. Δυσκολεύομαι αρκετά
e. Δυσκολεύομαι πολύ λίγο
f. Δεν έχω καμία δυσκολία

8. Όταν φοράτε μάσκα πόσο δυσκολεύεστε να διασχίσετε δρόμους;

a. Αναγκάζομαι να βγάζω τη μάσκα σε αυτή την περίπτωση
b. Σχεδόν δεν μπορώ να διασχίσω δρόμους χωρίς βοήθεια λόγω της όρασής μου και της χρήσης μάσκας
c. Δυσκολεύομαι πολύ
d. Δυσκολεύομαι αρκετά

e. Δυσκολεύομαι πολύ λίγο
f. Δεν έχω καμία δυσκολία

9. Όταν φοράτε μάσκα πόσο δυσκολεύεστε να διαβάσετε εφημερίδα ή ένα έντυπο κείμενο;

a. Αναγκάζομαι να βγάζω τη μάσκα σε αυτή την περίπτωση
b. Σχεδόν δεν διαβάζω εφημερίδα ή έντυπα κείμενα λόγω της όρασής μου και της χρήσης μάσκας
c. Δυσκολεύομαι πολύ
d. Δυσκολεύομαι αρκετά
e. Δυσκολεύομαι πολύ λίγο
f. Δεν έχω καμία δυσκολία

10. Όταν φοράτε μάσκα πόσο δυσκολεύεστε να διαβάσετε μηνύματα στο κινητό σας τηλέφωνο;

a. Αναγκάζομαι να βγάζω τη μάσκα σε αυτή την περίπτωση
b. Σχεδόν δεν διαβάζω μηνύματα στο κινητό μου τηλέφωνο λόγω της όρασής μου και της χρήσης μάσκας
c. Δυσκολεύομαι πολύ
d. Δυσκολεύομαι αρκετά
e. Δυσκολεύομαι πολύ λίγο
f. Δεν έχω καμία δυσκολία

11. Όταν φοράτε μάσκα πόσο δυσκολεύεστε να διαβάσετε ένα κείμενο στην οθόνη του υπολογιστή σας;

a. Αναγκάζομαι να βγάζω τη μάσκα σε αυτή την περίπτωση
b. Σχεδόν δεν διαβάζω κείμενα στην οθόνη του υπολογιστή μου λόγω της όρασής μου και της χρήσης μάσκας
c. Δυσκολεύομαι πολύ
d. Δυσκολεύομαι αρκετά
e. Δυσκολεύομαι πολύ λίγο
f. Δεν έχω καμία δυσκολία

12. Όταν φοράτε μάσκα νιώθετε ότι περιορίζεστε ως προς το είδος των δραστηριοτήτων που μπορείτε να κάνετε (πχ. Άσκηση, διασκέδαση, και άλλα);

a. Συμφωνώ απόλυτα
b. Μάλλον συμφωνώ
c. Ούτε συμφωνώ/ ούτε διαφωνώ
d. Μάλλον διαφωνώ
e. Σίγουρα διαφωνώ

13. Όταν φοράτε μάσκα νιώθετε ότι περιορίζονται οι δυνατότητες επαγγελματικής ή μαθησιακής εξέλιξής σας;

a. Συμφωνώ απόλυτα
b. Μάλλον συμφωνώ
c. Ούτε συμφωνώ / ούτε διαφωνώ
d. Μάλλον διαφωνώ
e. Σίγουρα διαφωνώ

14. Όταν φοράτε μάσκα νιώθετε ότι περιορίζονται οι κοινωνικές σας σχέσεις (με φίλους, συγγενείς ή άλλους ανθρώπους);

a. Συμφωνώ απόλυτα
b. Μάλλον συμφωνώ
c. Ούτε συμφωνώ / ούτε διαφωνώ
d. Μάλλον διαφωνώ
e. Σίγουρα διαφωνώ

15. Όταν φοράτε μάσκα νιώθετε ότι χρειάζεστε περισσότερη βοήθεια από κάποιο άλλο άνθρωπο (συγγενή, φίλο ή κοινωνική υπηρεσία) για να πάτε στον μπακάλη ή στο φούρνο ή στο σουπερμάρκετ;

a. Συμφωνώ απόλυτα
b. Μάλλον συμφωνώ
c. Ούτε συμφωνώ / ούτε διαφωνώ
d. Μάλλον διαφωνώ
e. Σίγουρα διαφωνώ

16. Όταν φοράτε μάσκα νιώθετε ότι χρειάζεστε περισσότερη βοήθεια από κάποιο άλλο άνθρωπο (συγγενή, φίλο ή κοινωνική υπηρεσία) για να πάτε στην τράπεζα ή σε κάποια άλλη δημόσια υπηρεσία;

a. Συμφωνώ απόλυτα
b. Μάλλον συμφωνώ
c. Ούτε συμφωνώ / ούτε διαφωνώ
d. Μάλλον διαφωνώ
e. Σίγουρα διαφωνώ

17. Η χρήση μάσκας με κάνει πιο νευρικό / οξύθυμο (συναισθηματικά φορτισμένο).

a. Συμφωνώ απόλυτα
b. Μάλλον συμφωνώ
c. Ούτε συμφωνώ / ούτε διαφωνώ
d. Μάλλον διαφωνώ
e. Σίγουρα διαφωνώ

18. Η χρήση μάσκας κάνει τα μάτια μου να τσούζουν περισσότερο από ότι πριν.

a. Συμφωνώ απόλυτα
b. Μάλλον συμφωνώ
c. Ούτε συμφωνώ / ούτε διαφωνώ
d. Μάλλον διαφωνώ
e. Σίγουρα διαφωνώ

19. Η χρήση μάσκας κάνει τα μάτια μου να τρέχουν δάκρυα περισσότερο από ότι πριν.

a. Συμφωνώ απόλυτα
b. Μάλλον συμφωνώ
c. Ούτε συμφωνώ / ούτε διαφωνώ
d. Μάλλον διαφωνώ
e. Σίγουρα διαφωνώ

20. Η χρήση μάσκας κάνει τα μάτια μου να με ενοχλούν περισσότερο από ότι πριν.

a. Συμφωνώ απόλυτα
b. Μάλλον συμφωνώ
c. Ούτε συμφωνώ / ούτε διαφωνώ
d. Μάλλον διαφωνώ
e. Σίγουρα διαφωνώ
