# Supplementary material for: Determinants of compliance to the facemask directive in Greece: A population study
Source: PLoS One. 2021 Mar 19;16(3):e0248929. doi: 10.1371/journal.pone.0248929 (PMC7978275; doi:10.1371/journal.pone.0248929)
Supplement: S2 File — (PDF) [file pone.0248929.s002.pdf]

DeMask-20 - Questionnaire for the evaluation of life quality of people using a facemask

Department of Ophthalmology, University Hospital of Alexandroupolis

Democritus University of Thrace

PART A

Initials of name(s): ______

Gender:

a. Male
b. Female

Year of birth: ______

Do you use spectacles?

a. No
b. Yes, for distance
c. Yes, for near
d. Yes, for both distance and near

Do you use contact lenses?

a. No
b. Yes, rarely
c. Yes, frequently
d. Yes, almost always

Do you belong to a vulnerable group*?

a. No
b. Yes
c. I don't know

*As vulnerable groups were defined people with age of 65 years or older, severe heart or respiratory disease, resistant hypertension, uncontrolled diabetes mellitus, severe neurological or neuromuscular disease, kidney or liver failure, high body mass index (BMI), cancer, immunodeficiency or pregnancy.

Do you know your distance vision (in both eyes)? Please note your vision with correction (with spectacles or contact lenses, if you use)

a. No
b. Less than 20/200
c. Between 20/200 and 20/63
d. Between 20/50 and 20/32
e. 20/25
f. Almost excellent (20/20)

Do you comply with the directive of Health Ministry regarding the use of facemasks during your daily activities?

a. No, never
b. No, almost never
c. Sometimes
d. Yes, almost always
e. Yes, always

<u>**PART B**</u>

The questions 1 & 2 refer to people who are obliged to wear a facemask while driving (e.g. public transport drivers) or people who choose to wear a facemask while driving for other reasons (e.g. belonging in a vulnerable group). If you are not belong to these groups please proceed to the 3rd question.

1. When you wear a facemask, how difficult is it to drive during the day in familiar places?

a. I need to remove my facemask in this case
b. I have almost stopped driving because of my vision and the use of a facemask
c. Great difficulty
d. Some difficulty
e. Little difficulty
f. No difficulty

2. When you wear a facemask, how difficult is it to drive at night in difficult circumstances or in unknown places?

a. I need to remove my facemask in this case
b. I have almost stopped driving at night in difficult circumstances or in unknown places because of my vision and the use of a facemask
c. Great difficulty
d. Some difficulty
e. Little difficulty
f. No difficulty

The questions 3, 4 and 5 refer to people who are obliged to wear a facemask in their working environment (e.g. healthcare workers, people working in restaurants etc) or people who choose to wear a facemask for other reasons (e.g. belonging in a vulnerable group). If you don't belong to these categories please proceed to the 6th question.

3. When you wear a facemask in your working environment, how difficult is it to read an official document?

a. I need to remove my facemask in this case
b. I have almost stopped reading official documents because of my vision and the use of a facemask
c. Great difficulty
d. Some difficulty
e. Little difficulty
f. No difficulty

4. When you wear a facemask in your working environment, how difficult is it to read a text on computer?

a. I need to remove my facemask in this case
b. I have almost stopped reading a text on computer because of my vision and the use of a facemask
c. Great difficulty
d. Some difficulty
e. Little difficulty
f. No difficulty

5. When you wear a facemask in your working environment, how difficult is it to collaborate with your co-workers?

a. I need to remove my facemask in this case
b. I have almost stopped collaborating with my co-workers because of my vision and the use of a facemask
c. Great difficulty
d. Some difficulty
e. Little difficulty
f. No difficulty

The following questions refer to all participants

6. When you wear a facemask, how difficult is it to recognize familiar people on the opposite sidewalk?

a. I need to remove my facemask in this case
b. I cannot recognize familiar people on the opposite sidewalk because of my vision and the use of a facemask
c. Great difficulty

d. Some difficulty
e. Little difficulty
f. No difficulty

7. When you wear a facemask, how difficult is it to read road or store signs?

a. I need to remove my facemask in this case
b. I cannot read road or store signs because of my vision and the use of a facemask
c. Great difficulty
d. Some difficulty
e. Little difficulty
f. No difficulty

8. When you wear a facemask, how difficult is it to cross the road?

a. I need to remove my facemask in this case
b. I cannot cross the road without assistance because of my vision and the use of a facemask
c. Great difficulty
d. Some difficulty
e. Little difficulty
f. No difficulty

9. When you wear a facemask, how difficult is it to read a newspaper or a printed text?

a. I need to remove my facemask in this case
b. I can hardly read a newspaper or a printed text because of my vision and the use of a facemask
c. Great difficulty
d. Some difficulty
e. Little difficulty
f. No difficulty

10. When you wear a facemask, how difficult is it to read messages on your mobile phone?

a. I need to remove my facemask in this case
b. I can hardly read messages on my mobile phone because of my vision and the use of a facemask
c. Great difficulty
d. Some difficulty
e. Little difficulty
f. No difficulty

11. When you wear a facemask, how difficult is it to read a text on your computer?

a. I need to remove my facemask in this case
b. I can hardly read texts on my computer because of my vision and the use of a facemask
c. Great difficulty
d. Some difficulty
e. Little difficulty
f. No difficulty

12. When you wear a facemask, do you feel that the type of activities you are able to do are limited (e.g. exercising, entertainment etc)?

a. Absolutely agree
b. Probably agree
c. Neither agree nor disagree
d. Probably disagree
e. Absolutely disagree

13. When you wear a facemask, do you feel that your professional or learning abilities are limited?

a. Absolutely agree
b. Probably agree
c. Neither agree nor disagree
d. Probably disagree
e. Absolutely disagree

14. When you wear a facemask, do you think that your social relationships are limited?

a. Absolutely agree
b. Probably agree
c. Neither agree nor disagree
d. Probably disagree
e. Absolutely disagree

15. When you wear a facemask, do you think that you need more assistance from other people (e.g. friends, relatives or social services) to go to the bakery or to the supermarket?

a. Absolutely agree
b. Probably agree
c. Neither agree nor disagree
d. Probably disagree
e. Absolutely disagree

16. When you wear a facemask do you think that you need more assistance from other people (e.g. friends, relatives or social services) to go to the bank or to a public service?

a. Absolutely agree
b. Probably agree
c. Neither agree nor disagree
d. Probably disagree
e. Absolutely disagree

17. The use of a facemask makes me more nervous (emotionally stressed).

a. Absolutely agree
b. Probably agree
c. Neither agree nor disagree
d. Probably disagree
e. Absolutely disagree

18. The use of a facemask makes my eyes stinging more than before.

a. Absolutely agree
b. Probably agree
c. Neither agree nor disagree
d. Probably disagree
e. Absolutely disagree

19. The use of a facemask makes my eyes more watery than before.

a. Absolutely agree
b. Probably agree
c. Neither agree nor disagree
d. Probably disagree
e. Absolutely disagree

20. The use of a facemask causes more discomfort to my eyes than before.

a. Absolutely agree
b. Probably agree
c. Neither agree nor disagree
d. Probably disagree
e. Absolutely disagree
